# The Monty Hall problem revisited: Autonomic arousal in an inverted version of the game

**DOI:** 10.1371/journal.pone.0192542

**Published:** 2018-03-26

**Authors:** Eduardo Massad, Paulo Cesar Costa dos Santos, Armando Freitas da Rocha, Edward J. N. Stupple

**Affiliations:** 1 School of Medicine, University of Sao Paulo, Sao Paulo, Brazil; 2 London School of Hygiene and Tropical Medicine, London, United Kingdom; 3 College of Life and Natural Sciences, University of Derby, Derby, United Kingdom; 4 School of Applied Mathematics, Fundacao Getulio Vargas, Rio de Janeiro, Brazil; 5 Paulista University, Sao Paulo, Brazil; 6 Institute of Advanced Studies, University of Sao Paulo, Sao Paulo, Brazil; Technion Israel Institute of Technology, ISRAEL

## Abstract

The asymmetry of autonomic arousal for potential losses and gains was assessed by the galvanic skin response (GSR) of participants playing classic and inverted versions of the Monty Hall problem (MHP). In both versions, the prize remained the same (a pen valued at £10 for the right answer), but in the modified version, prizes were received prior to choosing the door. Both experimental groups showed increased levels of GSR while completing the task, demonstrating increased autonomic arousal during the game. However, a robust difference in GSR was detected between classic and inverted versions of the MHP, thus demonstrating the differing autonomic arousal involved in deciding between the alternatives presented by the game. Participants experienced a stronger autonomic response when they could lose the prize than when they could win the prize. This experiment presents the first demonstration of this effect on the MHP. The stronger autonomic arousal for the inverted task may indicate a stronger emotional reaction and/or greater attentional focus than for the standard version of the task. These data demonstrate that potential losses increase arousal in more complex tasks than is typically shown.

## Introduction

A classic finding in psychology is that participants experience asymmetries in the intensity of their good and bad experiences across a wide range of domains, including personal relationships, emotions, rewards and punishments, and electrophysiological reactions [1]. Increased intensity for negative experiences has been suggested to extend to subjective responses to gains and losses [1, 2].More specifically, loss aversion is the phenomenon whereby changes that result in losses loom larger psychologically than do those that result in gains [2]. This bias regarding negative outcomes may also play a role in status-quo bias; that is, people tend to prefer the status quo because the potential for losses due to a change are more salient than the potential benefits. Several studies have also suggested that the important differences between values (prices) set by buyers and sellers, a finding called the “endowment effect” [2, 3], is due to loss aversion. The endowment effect has been typically explained by loss aversion in that sellers anticipate a potential loss of the object they own and compensate by inflating the price of the object due to loss aversion. Standard accounts of loss aversion show that losses are weighed more heavily than gains. However, this view has been challenged due to inconsistent findings, and loss aversion effects have not been generalized across all paradigms [4]. The attentional-based model of losses is an alternative account that proposes that increased attention and potentially improved performance occur when a task involves possible losses [4, 5]. Consistent with both loss aversion and the attentional-based model, participants have shown greater physiological arousal when experiencing negative outcomes. However, this increased arousal is not always accompanied by behavioral loss aversion, a finding that the attentional-based model is better equipped to explain.

These psychological effects have been shown to have physiological and neurophysiological bases [6, 7]. Indeed, greater arousal (exhibited by heart rate and pupil dilation measures) [5] and increased frontal cortical sensitivity [6] are shown for losses when compared to gains in a variety of tasks. However, even in the absence of behavioral loss aversion, losses have been shown to have distinct effects on arousal and frontal and cortical activation [7]. For instance, in situations in which decisions are constrained by reduced deliberation time, the loss-attention account predicts higher sensitivity (and higher physiological arousal) to relative values [8]. This increased sensitivity was observed even in settings in which participants typically show no loss aversion (e.g., decisions from experience with small amounts) [9].

The present study seeks to replicate the evidence of increased arousal in loss situations by using a novel task, the Monty Hall Problem (MHP), which is more cognitively complex than the tasks that have typically been used in previous studies [10].

The MHP, also called the Monty Hall Game or Dilemma [10], is a brainteaser that has sparked the imagination of philosophers, psychologists, economists, physicists, and cognitive scientists and has led all of them to leave their own mark on the problem [10]. The mathematical entertainer Martin Gardner called the MHP a “wonderfully confusing problem”[11],and the cognitive scientist Massimo Piattelli-Palmarini wrote, “No other statistical puzzle comes so close to fooling all the people all the time …”[12]. In a series of experiments, Granberg and Brown [13] tested people’s idiosyncrasies when confronted with this problem. They found that even after fifty trials in an iterative version of the game, people behaved in a characteristically irrational way, failing to see the solution. Many years later, Burns and Wieth [14] reviewed fifteen studies involving the standard version of the game and concluded that the failure to find the solution is a robust phenomenon that is consistent across cultures, wording presentation and educational level.

The MHP is a convenient test benchmark for assessing choice-induced autonomic arousal (which may be indicative of cognitive/attention load [15] and/or the emotional states of players [15]) measured by the physiological autonomous response of players in two versions of the game, as indicated by galvanic skin response (GSR).

The MHP has three steps:

1. The contestant is presented with three closed doors. Behind one there is a valuable prize (a car in the original game), and there are dummy prizes (goats) behind the two remaining doors.
2. The contestant chooses one of the three doors.
3. Monty Hall (the host of the game) opens one of the remaining doors, revealing a goat (he always knows which door contains which items and always opens a door with a goat first).
4. The contestant is offered the opportunity to stick with or switch from the original choice.

This version of the MHP is called "classic" in this paper.

The MHP sparked enormous controversy among mathematicians due to its counter-intuitive solution [12, 13]. The intuitive solution is that when one of the doors is opened without the prize, the contestant faces a new dilemma with only two doors and one prize. The chance of winning increases from 33.33% to 50%. There would be no advantage in switching from the original choice because the chance of finding the correct door would be 50%. This intuitive analysis, however, is incorrect because the door opened depends upon the first choice of the contestant and, as such, the winning door would not be opened. After opening a door without the prize, the contestant has new information, and switching is the optimal choice. The Bayesian formal solution demonstrates that the probability of winning improves from one-third to two-thirds when switching.

Emotional responses and cognitive/attentional load can trigger autonomous nervous system (ANS) responses such as increased heart rate, skin conductance, and pupil dilation [4]. Therefore, it can be difficult to determine whether cognition, emotion or both are implicated when physiological arousal is observed. Physiological responses are nonetheless useful objective indicators of the cognitive/attentional loads and emotional states participants experience during a task. It should be noted that in the time between when Monty Hall opened the door with the goat and the time the contestant had to decide whether to stick with or switch from his/her original choice, individuals would be expected to experience variable levels of attentional load and distinct emotional states [14–16].

We aim to examine the autonomic arousal produced by two versions of the MHP. Decision-making under a time constraint and varying emotional states is expected to trigger the ANS (i.e., the sympathetic branch) [17].The GSR is a robust physiological measure that is a standard measure of physiological arousal in the judgment and decision-making literature [18].Moreover, it is well suited as a process-tracing method because it offers a continuous and unobtrusive measure of cognitive and attentional processing.

In this work, the physiological arousal of participants is measured by their GSR in the classic version of the Monty Hall problem described above and in a variant. In both versions, the prize remains the same (a pen worth approximately£10for the right door and candy for the wrong doors), but in the latter, the contestant receives the prize at the beginning of the game, that is, before choosing the first door. A second door is then opened containing candy, and the opportunity to switch is offered. During the experiment, autonomic arousal is measured through the GSR test. In both versions, when the chosen door (or the eventual switch) contains the pen, the volunteer can take it with him/her. Otherwise, he/she leaves empty-handed (classic group) or must return it to the experimenter (inverted group).We selected pens as the prize to replicate the object participants received in several classic endowment effect experiments [2].

The cognitive demands of both versions of the task could be considered equivalent because the underlying logic is consistent in both scenarios. However, the attention-based account would predict increased on-task attention due to the potential for losses occurring. This may also increase sensitivity to the underlying structure of the task. There may also be differences in emotional responses between the tasks if the experience of a loss is more intense than the experience of a gain. We propose that the inverse version of the game may provoke a stronger emotional response in the participants, either because it is based on a prepayment or due to attentional load increasing because a loss is a possible outcome [2, 5, 19]. Thus, the objective of this project is to test the hypothesis that due to the differing states of arousal, the GSR levels of participants playing the inverse version of the MHP will be greater than that of those who play the classic version.

## Materials and methods

### Design

The GSR register is composed of amplitude, rise time, area under the curve (AUC), and the inclination of the linear fitted curve between the end of the latency and the maximum amplitude (also known as the velocity to reach the maximum, VRM). The GSR was recorded for two conditions: the classic and the inverse versions of the Monty Hall problem.

### Participants

Sixty students who were enrolled in the Computer Sciences undergraduate course at the Universidade Paulista (UNIP) in Sao Paulo, Brazil, were recruited(mean age = 22.4, SD = 3.9). The sample size, according to the methods described in [20] and [21], was thirty students in each group.

### Apparatus

The GSR sensor consisted of a data logger, flash memory, and a sensor for fully self-contained data gathering. The sensor sent data in a digital format to a computer in both .xls and.txt formats. The software adjusted the captured signal automatically and provided applications for presenting data, including tables, graphs, data analysis, double-axis set up, statistic operations, and mathematical operations. The sensor’s technical specifications were as follows: range and operation modes from 0 to 10.0 μS (0 to 65,279.0Arb); ADC resolution 16.0 bit; Resolution 1–10.0nS; and maximum sample rate (S/sec) of 100.0.

The SC software NeuLog (*NeuronSensors Network Technology*) measured the GSR of each volunteer during the experiment, and the generated data were automatically transferred to a Microsoft Excel spreadsheet (.xls) and text format (.txt).

**Fig 1:**
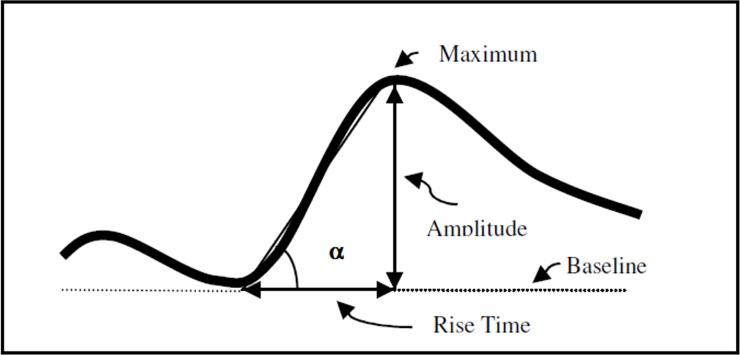
The components of a GSR autonomic response (Adapted from [18]). The VRM is shown by the inclination of the line from baseline to the maximum amplitude, which is the tangent of angleα.

The components of a typical autonomic response to the SC test are shown in Fig 1 below.

**Fig 2:**
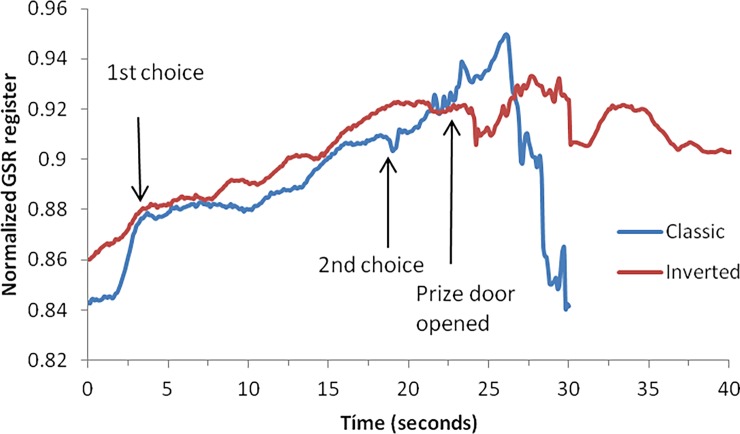
Normalized skin conductance register for the two groups. We interrupted the experiment when the skin conductance signal dropped back to the basal level, which occurred, on average, after 30 seconds for the classic version. In the inverse version, however, it did not return to the basal level after 40 seconds.

We measured the amplitude, rise time, AUC and the inclination of the linear fitted curve between the end of the latency and the maximum amplitude (VRM) (Fig 2).

The AUC was first normalized by dividing each individual value by the maximum number of observations in each group and was then calculated between the points represented by the end of the latency and the return to the basal level (or, at the end of the experiment, of SC response). The method used was the composite trapezoid rule [22] according to the equation $\int_{a}^{b}f(x)dx\approx \frac{\left(b-a\right)}{2N}\sum_{k=1}^{N}\left[f(x_{k+1})+f(x_{k})\right]$.

The amplitude measures the absolute values of GSR, a direct measure of arousal. The AUC, as the integral of GSR, represents total arousal, an indirect quantifier of physiological response. The rise time and the inclination of the linear fitted curve (velocity) are co-variates that indicate the magnitude of the physiological response (i.e., the shorter the time, the higher the speed at which the maximum amplitude of GSR is reached).

### Procedures

This work was reviewed and approved by the Institutional Review Board of the Universidade Paulista, where the study was conducted. Participants gave written informed consent prior to participating. All sixty participants in the study participated in a debriefing on the results one week after the experiments, and all received the pen, even those who did not choose the correct door.

Participants played one of two versions of the MHP in a computer environment. GSR measured physiological arousal in the classic version of the MHP (the player won the prize if and after he/she chose the correct door) and in the variant version (the player received the prize before the game and kept it if he/she chose the correct door). In both groups, the prize was the same (a pen worth approximately£10 for the right door and candy for the wrong doors), but in the latter, the volunteer received the prize before choosing a door. A door was then opened, revealing candy, and the volunteer was given an opportunity to switch. The winning door varied at random for each participant. In both versions, when the chosen door (or the eventual switch door) contained the pen, the volunteer could take it. Otherwise, the volunteer left with only the candy (classic group) or returned the prize to the experimenter (inverted group).

### Analytic strategy

After checking the normality of the distributions of the variables, amplitudes, rise times, AUCs, and the inclination of the linear fitted curve between the end of the latency and the maximum amplitude (VRM)using the Kolmogorov-Smirnoff test, the responses of the two groups were compared by either a t-test of independent samples (variables normally distributed) or a Mann-Whitney non-parametric test for independent samples (variables non-normally distributed).

All of the tests were one-tailed (the hypothesis was directional, that is, the inverted version group was expected to result in higher autonomic arousal levels than the classic version, and the significance level was assumed to be < 5% for statistical significance).

The statistical analyses were performed using the SPSS 18.0 software.

## Results

Crude data on SC of each participant are shown in S1, S2 and S3 Tables, with the results of the Classic, Inverted and Normalized Means experiments respectively.

Although the results for the MHP were not part of the objectives of this study, it is interesting that only four (two from each group) of the sixty participants (6.7%) switched doors. Of those who switched, two (50%) won the prize. Among those who did not switch, only seventeen (30.4%) won the prize. This is close to the classical result, but due to the small number of switchers, this difference was not significant (Fisher's exact test p = 0.68). Among the participants in each version of the game, two from each group switched doors, and one switcher from each group won the prize.

**Fig 3:**
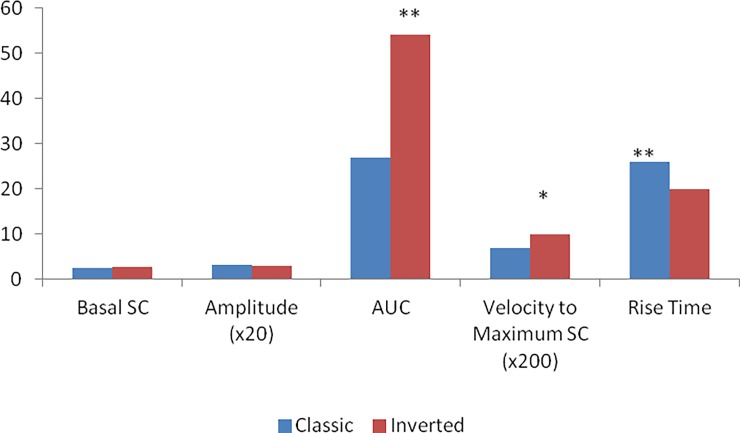
Mean values of the variables studied for the classic and inverted versions of the game. Mean amplitude and velocity to maximum SC were multiplied by 20 and 200, respectively, for better visualization (* p = 0.029, d = 0.24, ** p<0.0001, d = 0.90. ***p<0.0001, d = 0.86).

Fig 2 shows that participants in the inverted version of the game showed more physiological arousal (expressed by the AUC–“total” arousal) and reached the maximum GSR more quickly and earlier than their classical version counterparts. Fig 3 shows the normalized GSR register comparing the classic and the inverse versions of the game. After the prize door was opened, the classic version participants displayed a slight increase in GSR, probably due to the emotional effect of not gaining the prize, which dropped quickly to the basal level. Participants in the inverse version, in contrast, showed a slight drop in skin conductance, which rapidly increased after a few seconds; their register did not return to basal level even after the experiment terminated. The difference between the two registers in the interval between 22.5 sec and 27.5 sec was significantly different (Mann-Whitney U (n_1_ = 30; n_2_ = 30) = 536, Z = -6.202, p<0.001, Cohen’s d = 0.80).

Fig 3 shows the mean results of the variables studied in each experimental condition.

It can be noted from Fig 2 that although both the velocity to maximum GSR and the rise time to reach the maximum amplitude resulted in significant differences between the two experimental groups, the patterns observed were very similar. For the AUC, however, the results were very different, with a higher area observed in the inverse version group, implying a higher level of physiological arousal.Table 1 contains the descriptive statistics for the GSR measures as a function of the MHP condition.

**Table 1:** Descriptive statistics of the studied variables (N = 60).

Experimental Condition								
	Classic Version (N = 30)				Inverted Version (N = 30)			
	Range		Mean	SD	Range		Mean	SD
Variables	Min	Max			Min	Max		
**Basal SC (μS)**	**0.275**	**8.17**	**2.58**	**2.14**	**0.562**	**11.82**	**2.75**	**2.69**
**Amplitude (μS)**	**0.029**	**0.33**	**0.157**	**0.089**	**0.030**	**0.47**	**0.144**	**0.105**
**Area Under the SC Curve**	**22.17**	**29.5**	**26.85**	**1.90**	**44.86**	**58.60**	**54.19**	**4.00**
**Velocity to Max SC (μS/sec)**	**9E-04**	**0.35**	**0.035**	**0.072**	**1.7E-4**	**0.52**	**0.050**	**0.095**
**Rise Time (sec)**	**13.7**	**30.0**	**25.90**	**4.32**	**3.4**	**30.0**	**19.86**	**8.62**

After applying the Kolmogorov-Smirnov normality test, the appropriate independent samples’ comparative tests were applied. The basal skin conductance, amplitudes, and rise times were found to be normally distributed (crude data on the skin conductance of each volunteer are available in the S1, S2 and S3 Tables of the Supporting Information).)The areas below the SC curve and velocities to maximum SC were not normally distributed; these comparisons used a non-parametric test.

There were no significant differences between the classic and the inverted versions of the game with respect to the basal skin conductance (t (58) = 0.27, p = 0.394) and amplitude (t (58) = 0.509, p = 0.307). In contrast, the variables areas under the curve (Mann-Whitney U (n_1_ = 30; n_2_ = 30) = 465, Z = -6.653, p <0.001, one-tailed, d = 0.86), velocity to maximum GSR (Mann-Whitney U (n_1_ = 30; n_2_ = 30) = 322.5, Z = -1.888. p = 0.029, one tailed, d = 0.24)and rise time (t (58) = 3.426, p < .001, d = 0.9) showed significant differences between the classical and inverted versions of the game.

## Discussion

The results of this study reveal reliably higher levels of GSR for participants playing an inverse version of the MHP compared with the classic version. The velocity to maximum GSR, AUC and rise time were significantly different, with maximum GSR reached more quickly in the inverted version of the game. This finding supports the hypothesis that a loss-based or negative version of the task would produce greater autonomic arousal than the standard version of the task.

In both versions of the game, the same prize was at stake: a pen valued at £10.00 that the participants took with them if they chose the right door. In the inverted version, however, the participants received the prize before making the first choice (effectively a prepayment condition). Although these prizes initially appear equivalent, the prepayment literature, loss aversion and attentional model of loss suggest that these conditions are not equivalent, with participants preferring prepaid rewards, preferring to avoid losses, or increasing on-task attention [2, 3, 5]. The data show that the MHP with a prepaid reward had a stronger effect on the physiological arousal of the participants than did the prospect of eventually gaining the prize. This finding is consistent with the physiological arousal experienced by participants reflecting the perceived utility of the prize, but it could also indicate a cognitive load resulting from increased on-task attention [2, 5].

Although many psychological aspects of the MHP have been addressed in the literature (see, e.g., [23]), to the best of our knowledge, no study has addressed the physiological arousal induced by the uncertainties related to the decision-making demanded by the game. The present study, therefore, is the first to measure electro-dermal activity as an indication of physiological arousal related to both the classic and inverted versions of the MHP.

Our results can be interpreted as follows. In the first phase of the experiment, between time *t* = 0 and the opening of the first door, participants in both groups experienced increased GSRs due to the excitement related to the possibility of winning/keeping the prize and the attentional effort related to deciding which door to choose. After the first door was opened and participants were offered the opportunity to switch, the increase in the arousal observed in both groups of participants appeared to be related to the attentional load of decision-making under uncertainty. In the period between the second choice and the opening of the prize door, both groups continued to increase their arousal state in anticipation of the result. Participants in both groups subsequently increased their GSRs due to their reaction to the outcome of not winning/losing the prize. From this moment, however, the groups’ GSRs diverged. The members of the classic group experienced a marked decrease of their arousal state, but the inverted group’s GSRs remained high.

Our results indicate that losses of prepaid rewards increase physiological arousal relative to missing the opportunity to gain the prize. These findings are clearly consistent with Hochman and Yechiam[4], who found effects of pupil dilation and increased heart rate for losses even in situations where loss aversion behaviors were not observed [4].

The present study provides a valuable response to one of the open questions regarding the psychology of gains and losses as presented by Rick [24]–whether losses are experienced more intensely than gains–with our evidence clearly indicating that losses or tasks in which the potential for loss focuses attention are more arousing. Lejarraga and Hertwig show that this heightened response to losses also applies to exploratory searches [25]. In addition, DeMartino et al. [26] show that different frames in risky choices trigger different neural activations.

The variations in the electrodermal activity of the participants can be explained as indicative of differences in emotional responses because the underlying structure of the MHP tasks was identical and would be expected to entail identical cognitive demands. The findings are also consistent with the proposal of Yechiam and Hochman [5] that losses lead to greater attentional focus. Thus, although the present study cannot differentiate between the attention-based model, loss aversion and endowment effects, it provides a useful replication of increased autonomic arousal to losses with a novel and cognitively complex task.

The MHP is an interesting and reliable benchmark for studying physiological arousal in decision-making. Future studies should explore other aspects of the autonomic responses of players in several other designs. For instance, it is well known that people see the advantage of switching as increasingly obvious as the number of doors increase the cognitive demand of the task is reduced [27]. In accordance with the present study, Page [27] found that fewer than 12% of participants in the classic three-door version of the game switched. Therefore, although the attentional load of the task may have been demonstrated through the GSR measure, only a small proportion of the participants identified the optimal response (this did not differ across conditions). This performance level has been shown to increase to 50% switching in a ten-door version and 95% in a 100-door version. It can be predicted that arousal with the increasing number of doors would be proportionally reduced as the cognitive complexity of the task decreases. Thus, although the present data do not differentiate between emotional and cognitive sources of physiological arousal from the task, the impact of task complexity on autonomic arousal in the classic and inverted versions of the MHP warrants future investigation.

In conclusion, we detected robust differences in autonomous responses between the classic and the new inverted versions of the MHP game. We argue that the observed differences in physiological arousal between the two experimental groups reflected the potential loss of the prepaid prize in the inverted version and that this triggered a more pronounced autonomic response. This may be due to higher attentional load of the decision process when the task involves a loss. Our work presents a new experimental model that future investigations of losses versus gains may utilize.

## Supporting information

S1 Table S1 Table shows the crude data on skin conductance of each participants in the classical version of the MHG. (XLSX)

S2 Table S2 Table shows the crude data on skin conductance of each participants in the inverted version of the MHG. (XLSX)

S3 Table S3 Table shows the normalized means of skin conductance of each participants in the classical and inverted version of the MHG. (XLSX)
